# A scoping review of media campaign strategies used to reach populations living with or at high risk for Hepatitis C in high income countries to inform future national campaigns in the United Kingdom

**DOI:** 10.1186/s12879-023-08603-3

**Published:** 2023-09-26

**Authors:** David Etoori, Monica Desai, Sema Mandal, William Rosenberg, Caroline A Sabin

**Affiliations:** 1 Centre for Clinical Research, Epidemiology, Modelling and Evaluation (CREME), Institute for Global Health, UCL, Royal Free Campus, Rowland Hill Street, London, NW3 2PF UK; 2 National Institute for Health and Care Research (NIHR) Health Protection Research Unit (HPRU) in Blood-borne and Sexually Transmitted Infections at UCL in partnership with the UK Health Security Agency (UKHSA), Royal Free Campus, Rowland Hill Street, London, NW3 2PF UK; 3 Blood Safety, Hepatitis, Sexually Transmitted Infections and HIV Division, UK Health Security Agency, 61 Colindale Avenue, London, NW9 5EQ UK

**Keywords:** Hepatitis C, Media campaigns, High income countries

## Abstract

**Background:**

With the advent of direct acting antivirals, the World Health Organisation proposed eliminating Hepatitis C as a public health threat by 2030. To achieve this, countries need to diagnose, engage in care and treat their undiagnosed populations. This will require sensitisation campaigns. However previous media campaigns have had mixed impact. We conducted a scoping review to identify and understand the impact of previous Hepatitis C media campaigns. These findings could inform the delivery of future campaigns.

**Methods:**

We searched five electronic databases for published literature on media campaigns conducted for Hepatitis C awareness, testing, and treatment in Organisation for Economic Co-operation and Development (OECD) countries since 2010. Two independent reviewers screened citations for inclusion. Additionally, we spoke to stakeholders in the Hepatitis C field in the UK and conducted a Google search to identify any unpublished literature. A quantitative synthesis was conducted to identify targeted populations, strategies and media used, aims and impact of the campaigns.

**Results:**

A title and year of publication screening of 3815 citations resulted in 113 papers that had a full abstract screen. This left 50 full-text papers, 18 were included of which 9 (50%) were from Europe. 5 (27.8%) of campaigns targeted minority ethnicities, and 9 (50%) aimed to increase testing. A Google search identified 6 grey literature sources. Most campaigns were not evaluated for impact. Discussions with stakeholders identified several barriers to successful campaigns including lack of targeted messaging, stigmatising or accusatory messaging, and short-lived or intermittent campaign strategies.

**Conclusion:**

Future campaigns will likely need to be multifaceted and have multiple tailored interventions. Campaigns will need to be sizeable and robust, integrated into health systems and viewed as an ongoing service rather than one-offs.

**Supplementary Information:**

The online version contains supplementary material available at 10.1186/s12879-023-08603-3.

## Background

In 2016, the World Health Organisation (WHO) proposed eliminating Hepatitis C virus (HCV) as a public health threat by 2030 [1]. This was now possible given the paradigm shift in HCV treatment following the advent of highly effective direct-acting antivirals (DAAs) which cure 9 out of 10 people living with HCV [2]. In 2020, there were an estimated 56.8 million people living with HCV globally and approximately 255,000 deaths were attributable to HCV worldwide in the same year [3, 4]. As part of these efforts, the UK government has committed to the WHO strategy with the NHS in England having an ambition to eliminate HCV ahead of the 2030 goal [5, 6]. Part of this commitment involves sensitisation and awareness campaigns which are being supported by implementing partners.

In order to achieve elimination, defined by the WHO as a reduction to low levels in the incidence of and mortality due to HCV, it is important that those who are living with HCV in the community, but who are as yet undiagnosed, are supported to test for infection, understand their diagnosis, linked to care and supported to engage with treatment. In England, a third of people living with chronic Hepatitis C are currently injecting drugs and about 60–70% of people who inject drugs and who are chronically infected with HCV are unaware of their diagnosis. Many of those who are aware of their diagnosis have sub-optimal engagement with care. Between 2015 and 2020 only 65.3% of people who tested HCV RNA positive in the England had a subsequent record indicating treatment initiation and 45.8% achieved sustained virologic response (SVR). Additionally, 60% of people living with chronic Hepatitis C have a previous injection risk but a substantial population may be unaware of this risk. There is also a further 10% who may have other risks for acquiring Hepatitis C, including being born in a high prevalence country, or being a recipient of infected blood products [7]. All these disparate groups require support to test, and to link to, and remain engaged in, care if they are to experience the best health outcomes.

A health campaign is defined as any attempt to promote public health by raising awareness, stimulating adoption of new behaviours, encouraging an attitude change, or promoting new health interventions. Noar et al. propose the Audience-Channel-Message-Evaluation (ACME) framework to guide effective design implementation and evaluation of campaigns. In this framework, campaigns typically have an intended target audience and use specific channels i.e., traditional or digital media, which are chosen to maximise reach to the target audience. Message design is guided by target audience and behavioural theories and evaluation can take three forms i.e., formative, process, or outcome evaluation [8].

There have been various national and sub-national campaigns that have focussed on raising awareness, testing, and engagement with care [9]. The last nationwide Hepatitis C campaign in the UK was the “FaCe It” campaign, launched on 29 June 2004 alongside the *Hepatitis Action Plan for England* [10]. The campaign ran to May 2005 and included a public awareness campaign launched by the Chief Medical Officer (CMO) [11]. The campaign was targeted at the general public, people living with Hepatitis C, and healthcare professionals; the latter were targeted in advance of the general public so that they were prepared for a potential increase in enquiries about HCV. This campaign had little impact on testing and treatment at the time and was not evaluated making it difficult to gauge its long-term impact. Therefore, there is a need to understand what might have influenced its impact.

Targeted campaigns can support testing, linkage to care, and engagement. This review was conducted to understand the impact of previous campaigns and to inform the planning and delivery of any future campaigns to increase awareness and testing. In this review, we aim to synthesise all available information on previous HCV campaigns (i.e., to describe the number of campaigns, target populations, media strategies utilised, and major aims of the campaigns) in high income countries in order to understand best practices and reflect on the lessons learnt that may inform a future planned campaign. We positioned this review around high income countries because the pattern and epidemiology of HCV in these countries is most similar to that in the UK. A secondary objective of this review was to ascertain the effectiveness of these campaigns.

## Methods

### Review design

No protocol was registered for this review. The scoping review strategy was developed using the scoping review methodological framework proposed by Arksey and O’Malley and refined by the Joanna Briggs institute [12]. We used a Participants, Concepts, Context (PCC) framework to formulate the scoping review question, with *participants* defined as people living with or at high risk of acquiring HCV, the *concept* defined as media campaigns, and the *context* as high-income countries. The scoping review question was, “What media campaign strategies have been used to reach populations living with or at high risk for Hepatitis C in high-income countries?”

We then used the OVID MEDLINE database to search for medical subject heading (MeSH) terms that could be used synonymously with our participants, concepts and context (Additional file 1: Appendix 1). These terms were used to search relevant journal article electronic databases.

We also spoke to key stakeholders to learn if they had run any media campaigns or if they were aware of unpublished reports evaluating previous media campaigns. Finally, we conducted a Google search to identify any further grey literature or other online materials dealing with HCV media campaigns.

### Eligibility criteria

For the published literature, only studies published after 2010 were considered as we aimed to identify campaigns that had happened since the conclusion of the “FaCE It” campaign in the UK and since the advent of DAAs, a precursor to the shift towards Hepatitis C elimination [13]. Studies should originate from a high-income country as defined by Organisation for Economic Co-operation and Development (OECD) [14] (Table 1).

**Table 1:** Eligibility criteria

Criterion	Inclusion criteria	Exclusion criteria
**Concept**	- Studies, reports, and other communications on HCV media campaigns that report three components, the target population, the media strategy/channel of communication utilised, and the aim of the messaging of the campaign.	- Studies not reporting on the audience, channel, and message of HCV media campaigns. - Studies reporting on public health media campaigns not involving HCV. - Studies not reporting on a media campaign.
**Context**	- Campaigns aimed at the general public, people at high risk for HCV (e.g., PWID, people from countries with high HCV prevalence) or those living with HCV.	
**Language**	- Papers published in English.	- Papers published in any other language.
**Time period**	- Papers published from 1st January 2010 and onward. (Boceprevir was the first DAA to receive FDA approval in May 2011; 2010 was a pragmatic choice to cater for potential early adopters.)	- Papers published on or before 31st December 2009.
**Types of sources**	- Primary studies, study protocols and review, discussion, methods, and opinion papers reporting on HCV campaigns. - Grey literature including reports, theses, and official documents. - Information garnered from interviews with key HCV stakeholders in the UK.	- Non-professional sources (e.g., blogs).
**Geographic**	- Papers originating from high-income countries as defined by OECD.	- Research from low- and middle-income countries.

### Information sources and search strategy

Comprehensive literature searches were conducted using the five following electronic databases from inception until May 6, 2022: MEDLINE, PubMed, Scopus, Embase, and Web of Science. The final search strategy for MEDLINE can be found in Additional file 1: Appendix 2. Reference lists of included articles were also screened to identify any other studies of interest. We additionally searched for the term “Hep C media campaigns” in the Google search engine and examined the first 100 results.

Key HCV stakeholders in the UK were identified through consultation with UK Health Security Agency (UKHSA) researchers who had relevant experience with the UK HCV community. Identified stakeholders were asked to suggest any other expert sources we might have missed. Additionally, any stakeholder organisations identified through the scoping review Google search were also approached. These included both national and regional organisations (Table 2) as well as leading hepatitis researchers and hepatologists.; the insights of these people were collected via video call. While discussions were informal, we used a question checklist (Additional file 1: Appendix 3) to ensure consistency between the different interviews.

**Table 2:** Key stakeholder organisations approached for insights during the scoping review

Organisation	Role
**The National Health Service (NHSE)**	A publicly funded healthcare system in England.
**The Hepatitis C Trust**	A patient-led and patient-run charity organisation that has been campaigning on various issues related to Hepatitis C since 2001.
**The British Liver Trust**	A liver health charity supporting people affected by liver disease and liver cancer and working to improve liver health for the UK population.
**Forward Leeds**	Adult and young people’s alcohol and drug service in Leeds
**Hepatitis Scotland**	A national voluntary sector organisation funded by the Scottish government to help improve the response to viral hepatitis prevention, treatment and support.
**Gilead Sciences**	A research-based bio-pharmaceutical company focused on the discovery, development, and commercialisation of therapies for people with life-threatening diseases. Gilead has been working with the NHS to increase testing and treatment of Hepatitis C since 2015.

### Study selection process

We screened titles of search results from each electronic database to identify any potentially relevant articles, which were imported into Microsoft OneNote. The abstracts of potentially relevant articles were then reviewed by DE using the inclusion criteria. Full text articles were screened by DE, where possible, to abstract relevant information from the published articles. Articles where there was uncertainty for inclusion were checked by MD who determined their inclusion.

For the first 100 results of our Google search, we investigated any potentially relevant links. This included screening published and unpublished articles, visiting relevant websites, searching social media pages, and watching videos that might have any relevance. Grey literature was downloaded and links to any relevant websites, social media pages or videos were also saved to OneNote.

### Data items and collection process

For included articles, we extracted data on study characteristics (e.g., the year the study was conducted, country where the study was conducted). We also extracted campaign data based on the ACME framework [8] including which populations were targeted, what media was used, the scale of the campaign (e.g., national versus regional), the aim of the campaign, and how the impact of the campaign was measured. Where further information was warranted, we consulted study leads and coordinators by email. A Microsoft Excel spreadsheet was used to chart identified studies, their characteristics, and the metrics used to describe the campaigns. This spreadsheet was iteratively updated as more studies were identified.

### Analysis and synthesis of results

We grouped campaigns by target population, media strategy or channel used, campaign scale, campaign aim, and whether the campaign was evaluated. We report on counts and proportions of the different characteristics. We also summarised studies identified by source, and by country of origin.

## Results

### Literature search

**Fig. 1:**
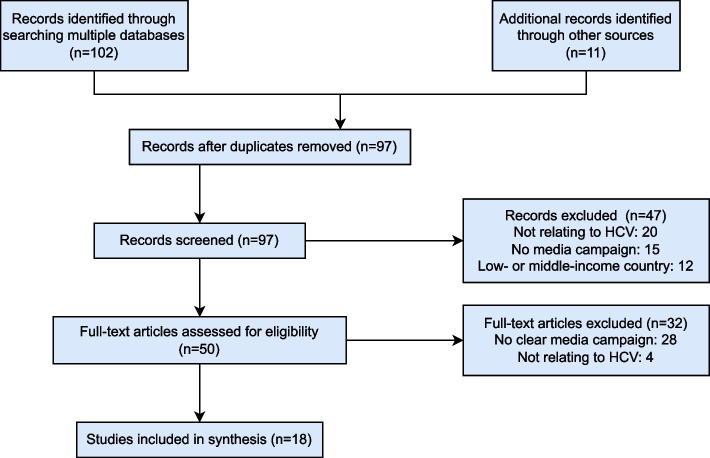
Flow chart showing details of published literature search

The literature search resulted in 3815 results (Fig. 1). After title screening, 113 citations were identified as potentially relevant and requiring abstract screening. Following abstract screening, 50 articles required a full text review with 18 articles included.

### Study characteristics

**Fig. 2:**
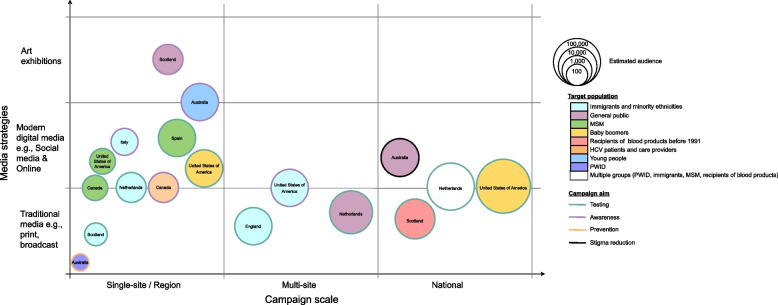
Parameters of the campaigns identified in published literature

The articles were published between 2010 and 2021 (Table 3). Of 18 included campaigns, each from a different campaign, 9 (50%) were from Europe and 5 (27.8%) targeted minority ethnicities and migrant populations in Europe. The most common aim was to increase HCV testing (50%). The scale of campaigns varied, with four (22.2%) studies reporting on nationwide campaigns, six (33.3%) on regional campaigns, two (11.1%) on multiple cities or sites, and six (33.3%) conducted in one city. Campaigns utilised traditional media (such as radio, broadcast television, and newsprint), social media, targeted internet adverts, and geosocial mobile apps particularly when targeting men who have sex with men (MSM). Two campaigns used art exhibits as a vehicle for education (Fig. 2).

**Table 3:** Characteristics of studies and campaigns

Study	Design characteristics and impact assessment	Study setting and dates	Target population	n	Media used and campaign strategy	Aim of the campaign
Helsper (2010) [15]	Cluster non-randomised controlled intervention in two regions. Analysis of testing trends.	Netherlands (2007–2008)	General public		Radio and newspaper advertisements and information distributed at public events. Primary care support programme consisting of distribution of educational materials to GPs.	Increase public awareness about HCV risk. Increase number of tests requested by GPs and number of HCV cases identified.
Uddin (2010) [16]	Testing offered on site. Participant follow-up.	Multiple cities in England. ()	Immigrant populations from India, Bangladesh, and Pakistan	4833	Internal adverts at mosques, public meetings. Recruited local religious leaders and community representatives as collaborators.	Establish prevalence of viral hepatitis in target migrants from South Asia.
Jafferbhoy (2012) [17]	Testing offered on site. Participant follow-up.	Dundee, Scotland (2009)	Immigrants from Pakistan.	170	Internal adverts at three mosques and one women’s centre, short talks. Community representatives were recruited as collaborators.	Increase HCV awareness. Encourage testing for HCV.
Richter (2012) [18]	Testing offered through mobile lab. Participant follow-up.	Arnhem, Netherlands. ()	Immigrants from Turkey.	709	A brochure, a poster, a video documentary and a website. Cooperation with community delegates. Materials disseminated through traditional media i.e., Turkish and Dutch newspapers, radio. Brochures were also distributed through Turkish shops and educational meetings were held at mosques and community centres.	To encourage testing for HCV and HBV.
Coughlin (2015) [19]	Materials developed using resources from the CDC. Evaluated for readability and cultural sensitivity using focus groups. Results not reported.	Multiple sites, USA ()	Adult African Americans.		Local radio, television, print and social media. Pamphlets and posters at community churches, markets, clinics, barbershops, hair salons, and laundromats. Education programme offered to interested healthcare providers.	Improve knowledge about HCV including risk factors, screening options, and availability of DAAs.
Treloar (2015) [20]	Focus groups with PWID and NSP staff.	Sydney, Australia. ()	PWID	21	Posters displayed at a needle and syringe program (NSP).	Hepatitis C prevention through reducing risky behaviour pertaining to injection drug use.
Jorgensen (2016) [21]	Nationwide campaign. Formative research for a year prior included literature reviews, surveys, and market analysis. Analysis of interactions with advertisements and website.	USA (2012)	Baby boomers	~ 200,000	Broadcast, out-of-home, digital, print, news media, and social media nationwide relying on donated time. Supplemented by targeted YouTube and Google advertisements and posts from CDC on Twitter and Facebook.	Encourage baby boomers to get tested and their healthcare providers to offer them tests.
Lampkin (2016) [22]	Created profiles and identified their purpose as health educators during conversations. Participant follow-up.	San Mateo county, USA. (2012–2014)	MSM	213	Passive outreach on geosocial mobile apps.	Determine acceptability of prevention messages through the app. Encourage testing and linkage to care.
Helsper (2017) [23]	Nationwide campaign. Targeted PWID campaign and narrowly focussed public intervention. Cost-effectiveness analysis	Multiple cities in the Netherlands. (2009–2010)	PWID and several risk groups in the general public including, immigrants, MSM, and recipients of blood products before 1992.		Health education through mass media and instruction of healthcare professionals. Included radio advertisements, website and internet banners, brochures, posters, and informative meetings.	To increase the number of HCV carriers identified.
Niebel (2017) [24]	Art pieces highlighted stigma issues affecting individuals with HCV. No formal evaluation was done.	Glasgow, Scotland. (2017)	General public		Artwork by affected individuals presented at an art exhibit.	Raise awareness about and reduce stigma.
Alarcón Gutiérrez (2018) [25]	Participant follow-up.	Barcelona, Spain. (2015–2016)	MSM	2656	Private messages on geosocial mobile apps.	Evaluate the acceptability and effectiveness of testing promotion through apps.
Buller-Taylor (2018) [26]	HCV course developed collaboratively with people affected by HCV. The course was administered through online and facilitated event presentations with electronic copies for participants without an internet connection. Pre- and post- survey.	British Columbia, Canada. (2014–2016)	HCV patients and HCV care providers.		Online course promoted a national network of partners, a nurse newsletter, and through a website.	Improve knowledge about HCV including risk factors, screening options, and availability of DAAs.
Eagle (2018) [27]	No formal evaluation.	Victoria, Australia. (2010- )	Young people		Interactive education sessions. Collaboratively developed photographic or video concepts. Entries are exhibited on a dedicated website, in public galleries, and shared on social media. Public votes for winning entries which receive a prize	Raise awareness about viral hepatitis.
Gilbert (2019) [28]	Analysis of new accounts on web-based testing service.	Vancouver, Canada (2015)	MSM	177	Location advertisements in print or video displayed in gay venues or events, advertisements on a queer news website, and advertisements on geosocial websites and apps	Evaluate the impact of the campaign on the use of a new internet-based testing service.
McLeod (2019) [29]	Nationwide campaign. Materials signposted participants to NHS website, a support helpline, and to GPs. Testing trend analysis.	Scotland (2015–2016)	Recipients of blood products before 1991.	22,842	Publicity from a national enquiry into tainted blood products. Information leaflets and posters distributed to GPs, pharmacies, dentists, and care homes.	Encourage HCV test uptake.
Plant (2020) [30]	Various testing pathways. Analysis of accounts on testing platform.	Los Angeles county, USA. (2015–2017)	Baby boomers	6919	A dedicated website. Targeted Facebook advertisements. A Facebook page was created where HCV information was posted.	To increase risk perception and lower barriers to testing.
Broady (2021) [31]	Nationwide campaign. Participants randomised to six groups. Pre- and post-survey.	Australia (2020)	General public	2010	Facebook advertisements for participant recruitment. Short video humanising populations living with BBVs to challenge stigmatising stereotypes.	Reduce stigma.
Frazzoni (2021) [32]	Pre- and post- survey	Emilia-Romagna, Italy. (2020)	People of Pakistani descent.	339	Dedicated website promoted through Facebook and mailing lists. Website hosted educational video.	Assess knowledge about HCV infection and effectiveness of the multimedia education strategy on improving knowledge.

### United Kingdom

Of four campaigns from the United Kingdom, 3 (75%) were from Scotland [17, 24, 29]. Two campaigns targeted migrant populations of south Asian ethnicity aiming to improve awareness and encourage testing for both HCV and HBV [16, 17]. Both campaigns recruited community leaders and representatives as collaborators. Campaigns used internal adverts at mosques and community centres. Additionally, educational talks were held during public meetings. Both studies offered on-site testing (results delivered by mail) and had high uptake of screening. Uddin et al. tested 4833 participants with 57 (1.2%) anti-HCV positive (55 HCV RNA positive) and 75 (1.6%) HBsAg positive [16]. Jafferbhoy et al. tested 170 individuals with 7 (4%) anti-HCV positive (5 HCV RNA positive), and 1 (0.6%) HBsAg positive [17]. One study targeted people who had received blood products before 1991 and built on a recent national enquiry in Scotland [29]. The campaign aimed to encourage testing in this population and used information leaflets and posters distributed to GPs, pharmacies, dentists, and care homes. Materials signposted individuals to an NHS website, support helpline, and their GP for further advice and testing. Impact was assessed by analysing trends in HCV testing in the year when the enquiry report was released (2015), and when the campaign was run (2016), finding a significantly higher number of tests in the week following the release of the report and the number mentioning blood transfusion for three weeks. These increases were not sustained following the awareness campaign. The final study described an art exhibit targeted to the general population in Glasgow which was aimed at reducing stigma [24]. HCV-affected individuals expressed their views on stigma and other facets of living with HCV using visual media under the guidance of experienced artists. Themes included, the rollercoaster of despair and hope, and the positive impacts of advancement in treatment through DAAs.

### United States

Two (50%) campaigns from the US targeted baby boomers and aimed to increase awareness and testing [21, 30]. One of these studies used targeted Facebook advertisements that linked directly to a testing website [30]. Additionally, a Facebook page was created where HCV information was posted. The page accumulated 49 followers between October 2015 and January 2017. Facebook advertisements reached 204,657 individuals with 8,547 (4.2%) clicks. Of 6919 unique testing website users, 2020 (29.2%) used the testing site locator, 48 (0.7%) accessed free testing. Of these 48, 1 (2.1%) was anti-HCV positive (RNA results were not reported). The second campaign used print, news, social and digital media nationwide relying on donated time [21]. These were supplemented by targeted paid advertisements using YouTube and Google directing participants to a website. Additionally, the CDC used Facebook and Twitter. Between 2012 and 2016, the campaign was shown in search results and other sites on the Google network 1.2 billion times. Google advertisements achieved a 0.4% click rate, YouTube advertisements were viewed 1.6 million times and the website was visited 200,000 times.

One study targeted African Americans in Memphis and surrounding areas aiming to encourage testing using advertisements on local radio, television, print, and social media [19]. This study was not evaluated and we could not find any further information on outcomes. The final study targeted MSM in California using social media with the aim of encouraging testing and linking participants to care [22]. The study reported that MSM from ethnic minorities were more likely to remain engaged with outreach staff.

### The Netherlands

In the Netherlands one study targeted immigrants of Turkish descent [18]. Study materials were developed by repurposing existing materials. Materials were disseminated through traditional media i.e., Turkish and Dutch newspapers, radio. Brochures were also distributed through Turkish shops and educational meetings were held at mosques and community centres. Testing was offered through a mobile laboratory. Screening uptake was high with 2 (0.3%) of 647 participants testing positive for anti-HCV and 18 (3.0%) for active HBV (HBsAg positive). Additionally, 25 (4%) participants were anti-HBc positive (no active HBV) with older participants, and first-generation migrants having higher anti-HBc prevalence. A review of hospital records revealed a prevalence of 0.7% for active HBV (higher in men) and 0.1% for active HCV (higher in women) among first generation migrants older than 24 years living in Arnhem. The most frequent risk factor mentioned was circumcision (method i.e., medical, or non-medical not specified).

A second campaign was run nationwide and targeted several risk groups (primarily PWID) [23]. It aimed to increase awareness, encourage testing, and increase the number of those infected with HCV identified. There were 299 additional positive anti-HCV tests resulting from the PWID intervention (257 had chronic HCV) compared to the control regions. For the public intervention, there was a 6.8% (95% CI: 6.2–7.4%) net increase in anti-HCV tests comparing the intervention and control regions during the intervention period. There was also a 5.9% (95% CI: 0.4–11.5%) net increase in the number of anti-HCV positive tests comparing intervention and control regions.

The final campaign used a similar strategy to the national campaign but was only run in one intervention region [15]. Number of tests requested increased from 57 to 172 in the intervention region compared to 86 to 118 in the control region (2.2 (95% CI: 1.5–3.3) times higher in the intervention region). There was a 2.6% (95% CI: − 0.7–5.8%) increase in anti-HCV cases identified in the intervention region compared to the control region. Positive tests were confirmed HCV RNA testing.

### Australia

Campaigns from Australia targeted PWID, [20] younger people, [27] and the general public [31]. The PWID campaign aimed to reduce higher risk behaviour around injecting including needle reuse with posters displayed at a needle and syringe program (NSP) site. The other two campaigns aimed to raise awareness and reduce stigma. The campaign targeted at younger people encouraged participants to develop video or photographic concepts which were exhibited on a dedicated website and in public galleries and shared on social media with a public vote for winning entries. The public campaign used targeted Facebook advertisements which signposted participants to educational materials and a survey.

### Canada

One campaign targeted MSM using location advertisements in print or video displayed in gay venues or events, advertisements on a queer news website, and advertisements on geosocial websites and apps [28]. The campaign aimed to evaluate the impact of advertisements on the use of a new internet-based testing service. During the study, 177 individuals created an account, with 83 (46.9%) being directed from advertisements from apps, 52 (29.4%) from the campaign website, 21 (11.9%) from location advertisements, and 20 (11.3%) from the news website.

The second campaign targeted HCV patients and care providers and aimed to reduce knowledge gaps [26]. The intervention utilised an online course promoted through a website, a nurse newsletter, and a national network of partners.

### Italy

The Italian campaign targeted people of Pakistani descent living in northern Italy [32]. The intervention consisted of an educational video on a dedicated website and aimed to improve knowledge about HCV. The website was promoted on Facebook and through a mailing list. Participants were administered pre- and post-video questionnaires to assess impact. Of 600 people contacted, 339 (56.5%) took part. Pre-video knowledge was low with 32% of participants unaware of HCV, 42% unaware of the potential for long-term HCV infection, and 14% aware of access to DAAs through health services. Only 67 participants answered the post-video interview with HCV awareness improving to 97%, awareness of long-term HCV infection to 99% and awareness of DAA access to 93%.

### Spain

The Spanish campaign was targeted at MSM and aimed to encourage testing for blood-borne viruses and sexually transmitted infections and to evaluate acceptability and effectiveness of testing promotion through geosocial mobile applications [25]. In total, 2656 individual messages were sent with 1019 responses (38.3%) (34 blocked or rejected the message and 139 gave indifferent responses). Of the 846 who responded favourably, 108 (12.8%) were interested in testing or vaccination, 258 (30.5%) stated that they planned to test elsewhere or had already tested, and 480 (56.7%) provided no information. Among 108 who were interested in attending a facility, 79 (73.2%) attended. Respondents were more likely to be older, to have been online when the message was sent or been online in the previous hour and using Grindr. None of 76 Hepatitis C tests performed was reactive.

### Stakeholder discussions

Discussions with Gilead involved the ‘Hep C Ki?’ campaign which targeted the British South Asian community and aimed to raise awareness and drive testing for HCV [33]. The campaign included a dedicated website, posters, leaflets, digital content, media and bus stop advertisements, and a partnership with social media influencers and community comedians. Additionally, five Operational Delivery Networks (ODNs) with community outreach were interviewed. Media advertisements reached over 700,000 people. However, testing uptake is not well understood as the campaign was not designed to measure this.

Discussions with Hepatitis Scotland involved two campaigns, Hep See, the art exhibit previous described [24] and #BeHepCFree [34]. The #BeHepCFree campaign aimed to raise awareness about DAAs and consisted of two strategies, two-minute animations based on recordings from interviews with people successfully treated using DAAs and a digital media campaign launched on World Hepatitis Day in 2020. Animations were posted on YouTube, Facebook and a dedicated website. The campaign was not officially evaluated.

### Grey literature

We identified a document comparing the ‘FaCe It’ campaign, a previous UK campaign to similar campaigns run in France around the same period [35]. The campaign utilised several resources including patient leaflets in multiple languages to provide basic information and answer the most important questions. Campaign posters highlighted and raised awareness about Hepatitis C. Essential information documents were distributed to assist healthcare professionals in offering testing to patients who may have been at risk of infection, a campaign booklet encouraged people to assess their individual risk, and other leaflets identified campaign media ambassadors (people living with Hepatitis C to share their experiences about living with HCV and speak to the media). Finally, a quick reference guide was developed for healthcare professionals to improve knowledge on the diagnosis and treatment of Hepatitis C. The authors identified several problems with the UK campaign including limited funding, lack of targeting, and stigmatising, accusatory messaging.

We also found a document published by Public Health England (PHE) that collated all resources developed by PHE to support an ongoing campaign to eliminate viral hepatitis [9]. This repository included posters, videos and banners for social media in multiple languages.

Additionally, we identified a review of screening campaigns for chronic viral hepatitis in migrant communities in Europe, [36] a study protocol for a community-based campaign focussed on assessing and improving HCV screening and treatment in Arizona, [37] and two documents summarising several campaigns undertaken in Australia [38, 39].

A Google search also identified web pages, news articles, social media pages, videos, and documents relating to several Hepatitis C media campaigns in multiple countries. These included campaigns reported earlier (e.g., Hep C Ki?, #BeHepCFree). Similarly, campaigns focused on raising awareness, stigma reduction, increasing testing demand, encouraging treatment seeking, and emphasising the ease of new Hepatitis C treatment. Target populations were diverse and similar to those identified through the published literature search and discussions with stakeholders including people living with Hepatitis C, south Asian populations in the UK, and baby boomers in the US. In the US, we also identified several media campaigns targeted at baby boomers run by pharmaceutical companies with the aim of boosting drug sales.

## Discussion

We conducted a scoping review on media campaigns utilised to reach people living with or at high risk of acquiring Hepatitis C finding 18 published studies and several other data sources including grey literature. We identified several campaigns with varying characteristics in terms of scale (national versus regional), target audience, methods used (traditional media, digital media, social media), strategy (targeted versus wide-ranging), aims, and evaluation methods.

Campaign aims included education, awareness, stigma reduction, increased testing, reengagement in care, increased treatment uptake, and harm reduction. Finally, campaign evaluation methods included media reach metrics, pre- post-test surveys, self-reported outcomes, and collection and analysis of primary data from various sources. There was also variability in measurement of impact. A systematic review of HIV campaigns reported similar findings with less than a third of campaigns included having a robust assessment component [40].

Where impact was reported, it was often not sustained owing to the intermittent or one-off nature of the campaigns. For example, in Scotland, following an enquiry into historic infected blood products, a testing campaign was launched but showed minimal impact that waned with time. Size, scale and dependable long-term funds appear to have the biggest impact on campaign effectiveness [41–44] with smaller scale campaigns with opt-in channels such as brochures showing smaller impacts [45]. Commercial entities recognise the importance of consistent and sustained advertising in maintaining brand awareness [46]. Furthermore, other studies have reported on waning effects after campaigns have concluded [47–51] and that funding cuts can have a similar impact [52]. As such, many studies suggest that repeated cycles are important for campaign effectiveness [53, 54] and that repetition improves impact [55–58]. This is likely because campaign effects may be modest but cumulative [59], and thus may be incremental with increased exposure [60, 61]. This was also emphasised in a report comparing the FaCe It campaign to a similar campaign in France where repeated messages had a greater and more sustained impact. Studies on smoking, nutrition, and cancer screening campaigns also suggest that these may be more effective when combined with other strategies such as community programmes [62], tailored reminder letters [63], and shifts in policy e.g., cigarette and sugary beverage taxes [44].

Discussions with hepatologists suggested that campaigns such as FaCe It, that were stigmatising, accusatory in tone, or sanctimonious were less likely to be impactful and may have adverse effects of discouraging people from coming forward for testing through fear of stigmatisation. This is contrary to findings from tobacco cessation campaigns where messages that evoked negative emotions of fear, disgust or sadness were more effective especially in populations with lower economic status [62, 64]. This finding is likely due to the nature of smoking which might be seen as a choice. The FaCe It campaign occurred in a different era where stigma was rife and DAAs were not yet available. There has since been a significant positive shift in framing of HCV and other blood-borne viruses which would likely be reflected in any potential campaigns. Data infrastructure has also improved which would make evaluation of the impact of any potential campaigns simpler.

Targeted campaigns appeared to have more impact and similar results have been demonstrated in several other studies [65]. This emphasises the need to know your epidemic and which groups are at highest risk before rolling out campaigns. For example, multiple campaigns targeting baby boomers in the United States reported high levels of engagement. Also, a Dutch study which took a two-pronged approach using both a general campaign and a campaign targeting PWID reported that the targeted campaign was more cost-effective. The use of behavioural insights and social marketing research also likely improve impact.

The potential for misinterpretation of campaign messages is an important consideration as emphasised by Treloar et al. [20]. Studies of smoking cessation campaigns in teenagers have reported opposite effects than were intended [66, 67] and studies of responsible drinking campaigns have shown that ambiguity can be detrimental and lead to little impact [68]. Clarity can be ensured by recruiting collaborators from target populations as a first point of contact. These collaborators can also be used to review materials for cultural sensitivity especially where minority groups are being targeted.

There still may be a need to explore new means of educating patients about tolerability of DAAs as misunderstandings still exist despite extensive media coverage. Despite a concerted effort to emphasise the superiority of DAA agents compared to interferon-based therapy, in terms of treatment ease, cure rates and tolerability, myths and outdated messages about treatment still exist among at-risk groups especially among those with direct experience with interferon and ribavirin treatment. This was emphasised by several campaigns including one by Forward Leeds where some participants mentioned not believing that the new treatment was better [69].

Finally, stakeholder discussions emphasised the need to prepare for the potential influx of patients requiring testing and treatment services. Parallel availability and access to key services is crucial to motivate action during campaigns [44]. For example, studies of breast cancer screening campaigns have shown that easy access to services improves impact [70] and that campaigns without organised screening services have little or no impact [71]. Three studies included in our review [16–18] utilised mobile testing which may have greater uptake than referral to GPs or laboratory but may not be scalable. Future campaigns will need to develop innovative strategies that balance scale, reach and expense, such as online testing portals which need to be evaluated for Hepatitis C. Furthermore, infrastructure put in place during the COVID-19 pandemic including new ways of BBV testing e.g., self-testing and home delivery of kits [72], delivery of HCV treatment directly to homes, clinical vans to ensure continuity of care, online provision of NSP, and enhanced community outreach [73] could be repurposed for this objective.

Future research could include a systematic review on the effectiveness of HCV media campaigns including in low- and middle-income countries. Our main goal was to collate evidence to inform a potential UK campaign, as such the review was positioned around high income countries which would likely have similar patterns and epidemiology of HCV. However, a systematic review could be used to identify evidence gaps and other research opportunities.

Our results will be of interest to other researchers and policy makers planning HCV testing or treatment campaigns. Future campaigns should be well targeted to achieve the desired impact. Campaigns should be clear on what population is being targeted and employ strategies to maximise reach in these groups. Future campaigns will likely need to be effectively broadcast depending on the population being targeted, salient and sustained to gain traction and have a lasting effect as it appears that one-off campaigns do not achieve any long-term impact. Future campaigns will need to think carefully about how impact will be measured. Evaluation plans need to be an integral part of campaign planning with easily measurable and reportable markers of impact. Future campaigns should employ nuanced messages emphasising different levels of risk and avoid stigmatising, accusatory messaging that risks discouraging people from engaging.

This study has several strengths. Firstly, the scoping review design allowed for a broad evaluation of multiple data sources, a first step in mapping the available information on this subject. Second is the use of several information sources i.e., published, and grey literature, stakeholder discussions.

Limitations include that scoping reviews inherently provide breadth rather than depth. However, this was appropriate as we aimed to collate available evidence rather than report on some ‘absolute’ effect of campaigns. Secondly, we only included studies published in English and only reported on campaigns from OECD countries. As such, our results are only generalisable to such settings which have specific HCV risk groups and epidemics.

## Conclusions

This review identified several media campaigns the majority of which targeted immigrants and ethnic minorities. Most of the campaigns were conducted in a single region/site and aimed to encourage testing. There was an equal mix of tradition media and modern digital media strategies employed to communicate campaign messages. Some campaign characteristics associated with success and failure were identified through discussions with stakeholders and could be used to inform future campaigns. Recommendations for future campaigns include the need to be multifaceted and have multiple tailored interventions [74]. Additionally, future campaigns should be sizeable and robust [75], integrated into health systems and viewed as an ongoing service [41], and shape how entertainment and news media interact with a complex public health issue [44] in order to have anticipated and sustained impact.

### Supplementary Information

**Additional file 1: Appendix 1.** Medline Search Strategy (Literature Search performed: May 6, 2022). **Appendix 2.** Table of participants, concepts, and context and MeSH terms used to search electronic databases. **Appendix 3.** Question checklist for informal discussions **Appendix 4**. PRISMA-ScR checklist.

## Data Availability

Data sharing is not applicable to this article as no datasets were generated or analysed during the current study.
