# Supplementary material for: A scoping review of media campaign strategies used to reach populations living with or at high risk for Hepatitis C in high income countries to inform future national campaigns in the United Kingdom
Source: BMC Infect Dis. 2023 Sep 26;23:629. doi: 10.1186/s12879-023-08603-3 (PMC10523688; doi:10.1186/s12879-023-08603-3)
Supplement: Supplementary file 1 — Additional file 1: Appendix 1. Medline Search Strategy (Literature Search performed: May 6, 2022). Appendix 2. Table of participants, concepts, and context and MeSH terms used to search electronic databases. Appendix 3. Question checklist for informal discussions Appendix 4. PRISMA-ScR checklist. [file 12879_2023_8603_MOESM1_ESM.docx]

**Appendix 1: Medline Search Strategy (Literature Search performed:**

**May 6, 2022)**

1. exp Hepatitis C/ or exp Blood-Borne Pathogens/ or Hepatitis C, Chronic/

2. exp Substance Abuse, Intravenous/ or exp Drug Users/ or exp Needle-Exchange Programs/ or exp Illicit Drugs/

3. exp Tattooing/

4. exp Unsafe Sex/

5. exp Homosexuality, Male/

6. exp “Ethnic and Racial Minorities”/

7. exp Homeless Youth/ or exp Homeless Persons/

8. exp Communications Media/ or exp Social Media/

9. exp Radio/

10. exp Television/

11. exp Internet/

12. exp Social Networking/

13. exp Mobile Applications/

14. exp “Marketing of Health Services”/ or exp Marketing/ or exp Social Marketing/

15. exp Advertising/

16. exp Health Education/

17. exp Health Promotion/

18. 1 or 2 or 3 or 4 or 5 or 6 or 7

19. 8 or 9 or 10 or 11 or 12 or 13

20. 14 or 15 or 16 or 17

21. 18 and 19 and 20 (356)

**Appendix 2: Table of participants, concepts, and context and MeSH terms used to search electronic databases.**

|  | Main concept | Alternate keywords |
| --- | --- | --- |
| Participants | Hepatitis C, high risk populations | Hepatitis viruses, hepatitis, hepatitis c, non-a, non-b hepatitis, viral, chronic hepatitis, blood-borne, intravenous drug user, drug addict*, drug abuse*, iv drug, people who inject drugs, substance abuse*, substance related*, substance use*, substance dependence, parenteral drug*, inject*, needle-exchange*, syringe exchange*, illicit drug*, illegal drug*, recreational drug*, tattoo*, body piercing, piercing*, high-risk sex, condomless sex/intercourse, unprotected sex, unsafe sex, MSM, ethnic and racial minorities, homeless persons |
| Concepts | Media campaign strategies  Advertising  Health education | Mass media, social media, print media, communications media, broadcast media, radio, television, twitter, Facebook, Instagram, dating apps, newspapers, publications, mobile app*, smartphone app*, internet, world wide web, web, cyber*, social network*, advertising*, marketing*, health education, awareness, digital |
| Context | High-income countries | Australia, united states, developed countries, industrialised countries/nations |

**Appendix 3: Question checklist for informal discussions**

- How would you describe your organisation's role in the Hep C community?
- Could I learn a bit more about what your roles are?
- In the past 10 years (since DAAs became available?), have you conducted any media campaigns around Hep C awareness, testing, and treatment?
- Could you give me a brief description of these campaigns (what was the primary media used, national or regional, etc)?
- What was the impact of these campaigns?
- Did you evaluate these campaigns?
- Any published evaluations you can share with me?
- What would you say were the main lessons learnt from this exercise, any mistakes you think are important to avoid?

**Appendix 4: PRISMA-ScR checklist**

| Item | Section | Included |
| --- | --- | --- |
| 1 | **Title:** Identify the report as a scoping review. | Y |
| 2 | **Abstract:** (Provide a structured summary that includes (as applicable) background, objectives, eligibility criteria, sources of evidence, charting methods, results, and conclusions that relate to the review questions and objectives.) | Y |
|  | **Introduction** |  |
| 3 | Rationale: Describe the rationale for the review in the context of what is already known. Explain why the review questions/objectives lend themselves to a scoping review approach. | Y |
| 4 | Objectives: Provide an explicit statement of the questions and objectives being addressed with reference to their key elements (e.g., population or participants, concepts, and context) or other relevant key elements used to conceptualize the review questions and/or objectives. | Y |
|  | **Methods** |  |
| 5 | Protocol and registration: Indicate whether a review protocol exists; state if and where it can be accessed (e.g., a Web address); and if available, provide registration information, including the registration number. | Y |
| 6 | Eligibility criteria: Specify characteristics of the sources of evidence used as eligibility criteria (e.g., years considered, language, and publication status), and provide a rationale. | Y |
| 7 | Information sources: Describe all information sources in the search (e.g., databases with dates of coverage and contact with authors to identify additional sources), as well as the date the most recent search was executed. | Y |
| 8 | Search: Present the full electronic search strategy for at least 1 database, including any limits used, such that it could be repeated. | Y |
| 9 | Selection of sources of evidence: State the process for selecting sources of evidence (i.e., screening and eligibility) included in the scoping review. | Y |
| 10 | Data charting/extraction process: Describe the methods of charting data from the included sources of evidence (e.g., calibrated forms or forms that have been tested by the team before their use, and whether data charting was done independently or in duplicate) and any processes for obtaining and confirming data from investigators. | Y |
| 11 | Data items: List and define all variables for which data were sought and any assumptions and simplifications made. | Y |
| 12 | Synthesis of results: Describe the methods of handling and summarizing the data that were charted. | Y |
|  | **Results** |  |
| 13 | Selection of sources of evidence: Give numbers of sources of evidence screened, assessed for eligibility, and included in the review, with reasons for exclusions at each stage, ideally using a flow diagram. | Y |
| 14 | Characteristics of sources of evidence: For each source of evidence, present characteristics for which data were charted and provide the citations. | Y |
| 15 | Results of individual sources of evidence: For each included source of evidence, present the relevant data that were charted that relate to the review questions and objectives. | Y |
| 16 | Synthesis of results: Summarize and/or present the charting results as they relate to the review questions and objectives. | Y |
|  | **Discussion** |  |
| 17 | Summary of evidence: Summarize the main results (including an overview of concepts, themes, and types of evidence available), link to the review questions and objectives, and consider the relevance to key groups. | Y |
| 18 | Limitations: Discuss the limitations of the scoping review process. | Y |
| 19 | Conclusions: Provide a general interpretation of the results with respect to the review questions and objectives, as well as potential implications and/or next steps. | Y |
| 20 | **Funding:** Describe sources of funding for the included sources of evidence, as well as sources of funding for the scoping review. Describe the role of the funders of the scoping review. | Y |
